# Study of the Expression of Sex-Determining Region Y-box 2 (SOX2) in Squamous Cell Carcinoma of the Cervix

**DOI:** 10.7759/cureus.110244

**Published:** 2026-06-04

**Authors:** Shalini Yadav, Shivangi Chauhan, Sharda Balani, Ashish Koshti, Reeni Malik

**Affiliations:** 1 Department of Pathology, Gandhi Medical College, Bhopal, IND; 2 Department of Pathology, Gandhi Medical College, Hamidia Hospital, Bhopal, IND

**Keywords:** cervical cancer, cervical squamous cell carcinoma, h-score, sox2, sox2 expression

## Abstract

Background: Cervical cancer remains a major health concern among women, particularly in low-resource settings. Sex-determining region Y-box 2 (SOX2), a transcription factor involved in cellular self-renewal and differentiation, has been implicated in tumor progression and malignancy.

Objective: This study aimed to evaluate and correlate the immunohistochemical expression of SOX2 across the spectrum of cervical squamous lesions and its association with clinicopathological parameters.

Methods: This cross-sectional observational study was conducted at Gandhi Medical College and Associated Hospitals, Bhopal, between May 2023 and October 2024. Eighty cervical specimens, including 63 biopsies and 17 hysterectomy samples, were classified as normal cervical epithelium (n = 20), cervical intraepithelial neoplasia (n = 20), and squamous cell carcinoma (SCC) (n = 40). SOX2 expression was evaluated by immunohistochemistry using a semiquantitative H-score based on staining intensity and percentage positivity. Statistical significance was assessed using the chi-square test with p < 0.05.

Results: SOX2 expression showed a significant progressive increase from normal cervical epithelium to malignant lesions (p = 0.0001). A higher proportion of SOX2-positive cases was observed in SCC cases (55%) than in premalignant (30%) and normal (0%) cases. A significant (p = 0.01) association was found between SOX2 expression and age groups and menopausal status (p = 0.01). No significant association was reported with SCC differentiation (p = 0.99) or International Federation of Gynecology and Obstetrics staging (p = 0.60).

Conclusion: SOX2 expression increases significantly with disease progression from normal epithelium to invasive carcinoma, suggesting its potential role as a biomarker for malignant transformation in cervical lesions. Further large-scale studies are required to establish its prognostic utility.

## Introduction

Cervical cancer ranks as the fourth most prevalent cancer among women worldwide and is strongly associated with persistent high-risk human papillomavirus (HPV) infection. Inadequate screening programs in low- and middle-income regions contribute substantially to its high mortality burden [1]. Emerging concepts propose that cancer stem cells (CSCs), a small population of tumor cells possessing self-renewal and strong tumor-forming capacity, contribute significantly to tumor growth and progression [2].

Sex-determining region Y-box 2 (SOX2) is a transcription factor important for maintaining stem cell pluripotency. Although primarily known for its role in embryogenesis, altered SOX2 expression has increasingly been implicated in squamous cell carcinomas (SCCs) [3].

Although p16 and Ki-67 are well-established immunohistochemical markers in cervical pathology, their diagnostic utility is mainly related to high-risk HPV-associated cell-cycle dysregulation and proliferative activity [1-3]. However, these markers do not directly reflect cancer stem-cell characteristics, cellular dedifferentiation, or tumor-initiating potential, which are increasingly recognized as important mechanisms in cervical carcinogenesis and disease progression. SOX2, a stemness-associated transcription factor, may therefore provide complementary biological information beyond conventional markers by identifying lesions with enhanced self-renewal capacity and malignant potential. Despite emerging evidence regarding SOX2 expression in cervical carcinoma, limited data are available on its expression pattern across the complete spectrum of cervical squamous lesions, particularly in the Indian population. Therefore, the present study was undertaken to evaluate SOX2 expression in nonmalignant cervical epithelium, premalignant cervical intraepithelial neoplasia (CIN), and invasive SCC, and to assess its association with clinicopathological parameters. This approach may help clarify whether SOX2 can serve as an adjunct biomarker for diagnosis, risk stratification, and future prognostic evaluation of cervical squamous lesions.

## Materials and methods

This cross-sectional observational study was conducted at Gandhi Medical College and Associated Hospitals, Bhopal, India, from May 2023 to October 2024, after obtaining approval from the Institutional Ethics Committee. A total of 80 patients with cervical specimens were included: 20 normal, 20 carcinoma in situ (CIN), and 40 SCC cases were obtained via biopsy (n = 63) and hysterectomy (n = 17).

The study received approval from the Institutional Ethics Committee of Gandhi Medical College, Bhopal (approval no: 18932/MC/IEC/2023; dated 09/05/2023). Written informed consent was obtained from all participants prior to enrolment.

Inclusion and exclusion criteria

Inclusion was limited to cervical squamous lesions, and patients with prior chemotherapy or radiotherapy and nonsquamous malignancies were excluded.

Histopathological analysis

All specimens were preserved in 10% neutral buffered formalin, processed routinely, and embedded in paraffin. Tissue sections of 3-5 µm thickness were stained with hematoxylin and eosin for histopathological assessment.

For immunohistochemistry, paraffin-embedded sections (3-5 µm) were mounted on poly-L-lysine-coated slides. Antigen retrieval was performed using citrate buffer (pH 6.0) in a pressure cooker for 15-20 minutes, followed by blocking of endogenous peroxidase activity with 3% hydrogen peroxide. Sections were then incubated with primary monoclonal anti-SOX2 antibody (clone EP103, rabbit monoclonal; PathnSitu Biotechnologies Pvt. Ltd., Hyderabad, India) at 1:100 dilution for 60 minutes at room temperature. Detection was performed using a horseradish peroxidase-conjugated secondary system with diaminobenzidine as the chromogen. Slides were counterstained with hematoxylin and mounted for microscopic evaluation. Known SOX2-positive SCC tissue served as the positive control, while negative controls were prepared by excluding the primary antibody.

Assessment of SOX2 expression was based on nuclear staining in epithelial or tumor cells using a semiquantitative scoring approach. The percentage of positive cells was graded as 0 (<10%), 1 (10%-25%), 2 (26%-50%), 3 (51%-75%), and 4 (>75%), whereas staining intensity was scored from 0 (negative) to 3 (strong). The final immunoreactive score was obtained by multiplying these two parameters, producing scores from 0 to 12. Based on total scores, cases were classified as negative (0), weakly positive (1-4), positive (5-8), or strongly positive (9-12). For statistical evaluation, negative and weakly positive cases were combined as low/negative expression, while positive and strongly positive cases were grouped as high/positive expression, considering that weak staining may reflect background reactivity rather than clinically relevant SOX2 overexpression.

Sample size justification

The sample size was estimated based on the primary objective of comparing SOX2 expression across nonmalignant cervical epithelium, premalignant lesions, and SCC. The main statistical test planned for this comparison was the chi-square test/Fisher’s exact test for categorical variables. Considering three diagnostic groups and two SOX2 expression categories, with an alpha error of 0.05, 80% power, and an expected moderate-to-large effect size for difference in SOX2 expression across diagnostic categories, the minimum required sample size was approximately 43 cases. In the present study, 80 cases were included, comprising 20 nonmalignant cervical tissues, 20 premalignant lesions, and 40 SCC cases. Based on the observed distribution of SOX2 expression across the three diagnostic groups, the achieved effect size was 0.47, and the post hoc statistical power was approximately 97%, indicating that the included sample size was adequate for detecting the primary association between SOX2 expression and histological diagnosis. However, subgroup analyses involving CIN grades, SCC differentiation, and International Federation of Gynecology and Obstetrics (FIGO) stage should be interpreted cautiously because of smaller numbers in individual subgroups.

Statistical analysis

Data analysis was performed using Statistical Package for the Social Sciences software version 20.0 (IBM Corp., Armonk, NY) after entry into Microsoft Excel (Microsoft Corporation, Redmond, WA). Continuous variables were presented as mean ± standard deviation, while categorical data were expressed as frequencies and percentages. The chi-square test was used to assess associations between categorical variables when assumptions were met, whereas Fisher’s exact test was applied for categories with expected cell counts below 5. Statistical significance was considered at p < 0.05.

## Results

Baseline characteristics of the study population

A total of 80 participants aged 30-79 years were included in the study. The largest proportion of patients was in the 40-49-year age group. Overall, the distribution of premenopausal and postmenopausal women was nearly comparable, while most patients were multiparous. Among SCC cases, postmenopausal women constituted the majority. The demographic and obstetric profile of the study population is summarized in Table 1.

**Table 1:** Demographic and obstetric profile of the patients Data are presented as frequencies (percentages); p value is not applicable for the descriptive table SCC: squamous cell carcinoma

Parameters	Frequency	Percentage (%)
Age groups (years)		
30-39	7	8.75
40-49	39	48.75
50-59	19	23.75
60-69	12	15
70-79	3	3.75
Total	80	100
Menstrual status		
Premenopause	41	51.25
Postmenopause	39	48.75
Total	80	100
Menstrual status of SCC patients		
Premenopause	14	35
Postmenopause	26	65
Total	40	100
Parity status		
Nulliparous	1	1.25
Primiparous	4	5
Multiparous	75	93.75
Total	80	100

The most common clinical complaint was white discharge, followed by abdominal pain, irregular bleeding, and postmenopausal bleeding. Tobacco use was uncommon, and a minority of patients reported oral contraceptive pill use. These clinical characteristics are presented in Table 2.

**Table 2:** Clinical complaints, tobacco use, and OCP intake in the patients Data are presented as frequencies (percentages); p value is not applicable for the descriptive table OCP: oral contraceptive pill

Parameters	Frequency	Percentage (%)
Clinical complaints		
White discharge	22	27.5
Abdominal pain	20	25
Irregular bleeding	18	22.5
Postmenopausal bleeding	17	21.25
Post coital bleeding	3	3.75
Total	80	100
Tobacco addiction		
No	77	96.25
Yes	3	3.75
Total	80	100
OCP intake		
No	66	82.5
Yes	14	17.5
Total	80	100

Histologically, the study included nonmalignant cervical epithelium, premalignant cervical lesions, and SCC. Among premalignant lesions, high-grade squamous intraepithelial lesion (HSIL) was more frequent than low-grade squamous intraepithelial lesion (LSIL). Among SCC cases, moderately differentiated SCC was the predominant histological grade, and FIGO stage III was the most common clinical stage. The detailed histological distribution and FIGO staging are shown in Table 3.

**Table 3:** Histological diagnosis and FIGO staging of patients Data are presented as frequency (percentage); p value is not applicable as the descriptive table CIN: cervical intraepithelial neoplasia, LSIL: low-grade squamous intraepithelial lesion, HSIL: high-grade squamous intraepithelial lesion, SCC: squamous cell carcinoma, FIGO: International Federation of Gynecology and Obstetrics

Parameters	Frequency	Percentage (%)
Histological diagnosis		
Nonmalignant	20	25
Premalignant	20	25
SCC	40	50
Total	80	100
Premalignant lesions		
LSIL (7, 35%)		
CIN I	7	35
HSIL (13, 65%)		
CIN II	10	50
CIN III	3	15
Total	20	100
SCC differentiation		
Well differentiated	8	20
Moderately differentiated	28	70
Poorly differentiated	4	10
Total	40	100
FIGO staging		
I	3	7.5
II	13	32.5
III	16	40
IV	8	20
Total	40	100

No statistically significant association was observed between age group and SCC differentiation (p = 0.45), as shown in Table 4. SOX2 histological score showed a significant association with histological diagnosis, with increasing expression from nonmalignant cervical epithelium to premalignant lesions and SCC (p = 0.0002). However, SOX2 score was not significantly associated with individual CIN categories (p = 0.45) or SCC histological grade (p = 0.39), as shown in Table 5.

**Table 4:** Comparison of SCC differentiation with age groups Data are presented as frequency (percentage); Fisher’s exact test was applied; p < 0.05 was considered statistically significant SCC: squamous cell carcinoma

Age groups (years)	SCC differentiation			p value
	Well differentiated	Moderately differentiated	Poorly differentiated	
30-39	1 (50%)	1 (50%)	0 (0.0%)	0.45
40-49	1 (7.14%)	10 (71.43%)	3 (21.43%)	
50-59	4 (36.36%)	7 (63.64%)	0 (0.0%)	
60-69	2 (18.18%)	8 (72.73%)	1 (9.09%)	
70-79	0 (0.0%)	2 (100%)	0 (0.0%)	

**Table 5:** Correlation of SOX2 histological score and histological diagnosis, CIN categories, and histological grades of SCCs Data are presented as frequency (percentage); Fisher’s exact test was applied; p < 0.05 was considered statistically significant SOX2: sex-determining region Y-box 2, CIN: cervical intraepithelial neoplasia, SCC: squamous cell carcinoma

Parameters	SOX2 histological score				p value
	0	1-4	5-8	9-12	
Histological diagnosis					
Nonmalignant	6 (30)	14 (70)	0 (0.0)	0 (0.0)	0.0002
Premalignant	1 (5)	13 (65)	5 (25)	1 (5)	
SCC	3 (7.5)	15 (37.5)	8 (20)	14 (35)	
CIN					
CIN I	1 (14.29)	6 (85.71)	0 (0.0)	0 (0.0)	0.45
CIN II	0 (0.0)	6 (60)	3 (30)	1 (10)	
CIN III	0 (0.0)	1 (33.33)	2 (66.67)	0 (0.0)	
SCC histological grades					
Well differentiated	2 (25)	1 (12.5)	3 (37.5)	2 (25)	0.39
Moderately differentiated	1 (3.57)	12 (42.86)	5 (17.86)	10 (35.71)	
Poorly differentiated	0 (0.0)	2 (50)	0 (0.0)	2 (50)	

When SOX2 expression was categorized as negative/low versus positive/high expression, a statistically significant association was observed with age group in the overall study population (p = 0.01), menopausal status in all cases (p = 0.01), and histological diagnosis (p = 0.0001). SOX2 positivity was highest among SCC cases compared with premalignant and nonmalignant lesions. However, no significant association was observed between SOX2 expression and age group among SCC cases (p = 0.42), menopausal status among SCC cases (p = 0.07), parity (p = 0.23), oral contraceptive pill intake (p = 0.51), CIN category (p = 0.24), SCC histological grade (p = 0.99), or SCC clinical stage (p = 0.60). These associations are summarized in Table 6.

**Table 6:** Comparison of SOX2 expression with various demographical, clinical, and histological parameters Data are presented as frequency (percentage); Fisher’s exact test was applied; p < 0.05 was considered statistically significant SOX2: sex-determining region Y-box 2, CIN: cervical intraepithelial neoplasia, SCC: squamous cell carcinoma, OCP: oral contraceptive pill

Parameters	SOX2 expression		p value
	Negative	Positive	
Age groups (years) (all cases)			
30-39	7 (100)	0 (0.0)	0.01
40-49	29 (74.36)	10 (25.64)	
50-59	10 (52.63)	9 (47.37)	
60-69	4 (33.33)	8 (66.67)	
70-79	2 (66.67)	1 (33.33)	
Age groups (years) (SCC cases)			
30-39	1 (50)	1 (50)	0.42
40-49	9 (64.5)	5 (35.7)	
50-59	4 (36.3)	7 (63.6)	
60-69	3 (27.2)	8 (72.7)	
70-79	1 (50)	1 (50)	
Menstrual status (all cases)			
Premenopause	32 (78.05)	9 (21.95)	0.01
Postmenopause	20 (51.28)	19 (48.72)	
Menstrual status (SCC cases)			
Premenopause	9 (64.29)	5 (35.71)	0.07
Postmenopause	9 (34.62)	17 (65.38)	
Parity status			
Nulliparous	1 (100)	0 (0.0)	0.23
Primiparous	4 (100)	0 (0.0)	
Multiparous	47 (62.67)	28 (37.33)	
OCP intake			
No	44 (66.67)	22 (33.33)	0.51
Yes	8 (57.14)	6 (42.86)	
Histological diagnosis			
Nonmalignant	20 (100)	0 (0.0)	0.0001
Premalignant	14 (70)	6 (30)	
SCC	18 (45)	22 (55)	
CIN			
CIN I	7 (100)	0 (0.0)	0.24
CIN II	6 (60)	4 (40)	
CIN III	1 (33.33)	2 (66.67)	
SCC histological grades			
Well differentiated	3 (37.50)	5 (62.50)	0.99
Moderately differentiated	13 (46.43)	15 (53.57)	
Poorly differentiated	2 (50)	2 (50)	
SCC clinical staging			
I-II	8 (50)	8 (50)	0.60
III-IV	10 (41.67)	14 (58.33)	

## Discussion

A total of 80 cases were included in this study, out of which 20 (25%) patients had normal cervix, 20 (25%) had premalignant lesions, and 40 (50%) were diagnosed with cervical SCCs. SOX2, a transcription factor belonging to the SOX family, is essential for regulating embryonic development, stem cell maintenance, and cellular differentiation. SOX2 also plays a role in tumorigenesis and cancer progression by promoting cell proliferation, invasion, and resistance to apoptosis. This comprehensive analysis provides insights into the role of SOX2 as a potential biomarker in the pathogenesis and progression of cervical neoplastic lesions.

The present study was designed to address an important gap in cervical lesion assessment. While p16 and Ki-67 are widely used to support the diagnosis and grading of HPV-associated squamous intraepithelial lesions, they primarily indicate viral oncogene-driven cell-cycle alteration and proliferative activity. In contrast, SOX2 reflects stemness-related biology, which may be involved in lesion progression, tumor initiation, and malignant transformation. Therefore, evaluation of SOX2 may provide additional information that is not fully captured by conventional biomarkers. By assessing SOX2 expression across nonmalignant, premalignant, and malignant cervical squamous lesions, the present study adds to existing evidence by demonstrating a progressive increase in SOX2 expression with disease severity and supporting its potential role as an adjunct marker rather than a replacement for established biomarkers.

In the current study, the highest proportion of cervical SCC cases was observed in the 40-49 years age group, comprising 35% of patients. The mean age among SCC patients was 53.12 years, with most cases occurring after 40 years of age. These findings align with observations reported by Dayalan and Raghavan [4] and Thakur et al. [5]. Thakur et al. [5] documented 45-54 years as the predominant age group for cervical SCC, with a mean age of 48.1 ± 5.4 years, whereas Dayalan and Raghavan [4] reported a greater incidence among women aged 46-55 years. Comparable findings have also been noted by Chang et al. [6], Yang et al. [7], and Ji et al. [8], suggesting that cervical SCC commonly presents during the fifth decade of life. All these studies support the trend of increased cervical SCC incidence with age. The finding of higher incidence of cervical SCCs in the older age group, particularly >40 years could be due to long latency period between HPV infection and cancer development, decline in immune surveillance with increasing age, atrophy and decreased cellular regeneration in cervical epithelium secondary to postmenopausal hormonal changes, cumulative exposure to risk factors like smoking, immune-suppression, high parity and poor genital hygiene, and missed opportunities for cervical diseases screening. These findings highlight the necessity of early intervention and regular screening in women approaching this age group to facilitate early detection and improve prognosis [9-11].

In contrast, Dahiya et al. [12] reported a mean age of 52.28 ± 11.29 years, and Pandey et al. [13] observed an age range of 51-60 years, indicating a wider age distribution that includes women from their early 40s to early 60s. The wider age distribution in these studies might be attributed to regional, genetic, or socioeconomic differences, or to variations in screening practices and population characteristics.

In the present study, SOX2 positivity was observed in 62.5% of patients below 49 years of age and in 33.3% of those above 49 years of age, although the difference was not statistically significant (p = 0.42). Similar results have been documented in earlier studies. Xu et al. [14] reported SOX2 positivity rates of 75.7% in patients younger than 45 years and 71% in older patients, without a significant association (p = 0.65). Likewise, Yang et al. [7] found positivity rates of 77.8% and 71.4% among patients below and above 42 years, respectively, with no statistically significant difference (p = 0.58). These findings suggest that SOX2 expression is not significantly associated with age, despite minor variations in positivity rates across age groups. Observed differences between studies may be attributable to variations in sample size, demographic profiles, or study methodology.

Overall, we observed a significant association between SOX2-positive expression and postmenopausal females compared with premenopausal females (p = 0.01). Also, most (64.29%) premenopausal SCC patients were negative for SOX2 expression, whereas most (75.38%) postmenopausal SCC patients were positive for SOX2 expression; however, there was no significant association between menopausal status and SOX2 expression (p = 0.07). Higher SOX2 expression in postmenopausal women could be mainly due to hormonal changes, especially the decline in estrogen levels after menopause. This hormonal shift may lead to upregulation of the SOX2 gene, which is linked to CSC activity and tumor progression. In cervical SCC, high SOX2 levels are associated with aggressive behavior, poor prognosis, and reduced tumor suppression (e.g., via p16INK4A pathways) in this demographic. Thus, postmenopausal hormonal changes may indirectly enhance SOX2 expression, contributing to worse cancer outcomes [15-17].

In the present study, 25% of cases were normal, 25% were premalignant, and 50% were cervical SCC, as determined by histological analysis. All cases were assessed for SOX2 expression. In the present study, a few nonmalignant cervical tissues showed only weak SOX2 immunoreactivity, mainly limited to the basal/parabasal epithelial layers. However, none of the nonmalignant cases demonstrated high/positive SOX2 expression based on the predefined scoring criteria. This weak basal staining may represent physiological expression in reserve or progenitor-like epithelial cells rather than true pathological overexpression. In contrast, premalignant and malignant lesions showed progressively increased SOX2 expression, supporting its possible role in cervical squamous carcinogenesis. In CIN1 lesions, positivity was primarily restricted to the basal and parabasal layers with extension into the intermediate layers. In contrast, CIN2 and CIN3 lesions showed SOX2 expression extending to the superficial epithelial layers, accompanied by increased staining intensity compared with CIN1. In SCCs, the staining pattern was complex, with some areas showing diffuse expression while others showed focal or reduced expression. In some cases, SOX2 expression was localized to specific areas within the tumor, suggesting a role in the development and progression of SCCs. A significant association was observed, with increasing SOX2 expression from normal to malignant tissues (p = 0.0001).

Dayalan and Raghavan [4] included 10% normal tissue, 41% premalignant, and 19% SCC cases. They found higher SOX2 expression in the basal layer of the normal cervix. Despite some variation in the percentages, the overall pattern of increasing SOX2 expression with disease severity remains consistent, emphasizing its possible role in tumor progression (p = 0.0003). Cai et al. [18] studied SOX2 expression in 11.6% of normal cervix, 41.8% premalignant lesions, and 46.5% SCC cases. They observed a p value of 0.0001 for these cases. Their findings closely align with our results, supporting the notion that SOX2 expression increases as cervical lesions advance from benign to malignant. Yang et al. [7] and Kim et al. [15] also supported the above findings.

The above findings could be due to SOX2's role in promoting stemness, cellular dedifferentiation, and tumor progression. As a transcriptional factor essential for maintaining stem cell properties, SOX2 is increasingly expressed as cervical lesions advance, contributing to uncontrolled proliferation and the development of CSC-like traits that support tumor growth and therapy resistance. This upregulation is often driven by genomic proliferation of the SOX2 locus on chromosome 3q26 and further enhanced by high-risk HPV infections, particularly HPV16 and HPV18, which interact with and upregulate SOX2 expression. Consequently, various studies have found that SOX2 not only plays a key role in cervical carcinogenesis but also serves as a valuable biomarker to distinguish high-grade lesions and invasive carcinoma from benign or low-grade conditions [19,20].

In the present study, SOX2 expression was higher in CIN III compared to CIN II and normal cervical tissue, although no significant difference was observed among individual CIN grades. However, SOX2 expression was significantly greater in HSIL than in LSIL lesions (p = 0.03). Similar findings were reported by Moshi et al. [19], who demonstrated progressive expansion of SOX2 expression from the basal/parabasal layers toward the intermediate and superficial epithelial compartments during early CIN progression. They also described an unusual expression pattern in CIN II and CIN III lesions, characterized by reduced or absent SOX2 expression in basal layers with variable positivity in intermediate and superficial layers. Increased SOX2 expression in HSIL compared with LSIL has likewise been reported by Dayalan and Raghavan [4] (p = 0.000), Kim et al. [15], and Cai et al. [18] (p = 0.01). High SOX2 expression in HSIL was in agreement with the fact that SOX2 regulates cell proliferation and the undifferentiated state, and its overexpression contributes to uncontrolled proliferation and maturation failure in HSIL. High-risk HPV, the main cause of CIN, can stabilize and enhance SOX2 expression. Additionally, HSIL has more cells with stem cell-like properties, reflected in higher SOX2 levels, and this increase is associated with a greater risk of progression to invasive cervical cancer [19].

In the present study, moderately differentiated SCC was the most common histological grade. Although SOX2 positivity was observed across all grades of SCC, no statistically significant association was found between SOX2 expression and tumor differentiation. This finding suggests that SOX2 expression may not directly parallel conventional histomorphological grading in cervical SCC. One possible explanation is that SOX2 primarily reflects stemness-related biology and early malignant transformation rather than the degree of squamous differentiation seen on routine histology. Tumor grade is a morphological parameter, whereas SOX2 expression reflects molecular regulation of self-renewal, dedifferentiation, and tumor-initiating potential; therefore, discordance between the two may occur. Similar findings were reported by Dayalan and Raghavan [4] and Yang et al. [7], who also did not observe a significant association between SOX2 expression and SCC differentiation. In contrast, Chang et al. [6] reported higher SOX2 expression in poorly differentiated SCC and found a significant association with tumor differentiation. These conflicting findings may be related to differences in sample size, antibody clone, scoring method, cutoff values for positivity, HPV-related molecular background, and intratumoral heterogeneity. In the present study, the small number of poorly differentiated SCC cases may also have limited the ability to detect a statistically significant grade-wise difference.

The present study showed a higher proportion of SOX2 positivity in FIGO stage III-IV disease compared with stage I-II disease; however, this difference was not statistically significant. This finding should be interpreted cautiously. FIGO staging is primarily an anatomical and clinical staging system based on tumor extent, local invasion, and spread, whereas SOX2 is a molecular marker associated with stemness, self-renewal, and tumor cell plasticity. Therefore, SOX2 expression may be more closely related to early carcinogenic transformation and biological aggressiveness than to anatomical stage at presentation. Previous studies have also shown inconsistent findings. Yang et al. [7], Ji et al. [8], and Kim et al. [15] did not report a significant association between SOX2 expression and FIGO stage, supporting the observation that SOX2 may not consistently increase with clinical stage. In contrast, studies reporting stronger associations between SOX2 and advanced clinicopathological features suggest that SOX2 may contribute to aggressive tumor behavior in selected cohorts. Such discrepancies may be explained by differences in cohort size, the distribution of early and advanced stages, inclusion criteria, staining protocols, scoring thresholds, and the absence or presence of correlation with HPV status and other biomarkers. In the present study, the limited number of early-stage cases and the predominance of advanced-stage disease may have reduced the discriminatory power of FIGO-based subgroup analysis.

The results of this study suggest that SOX2 could serve as a promising biomarker for distinguishing high-grade cervical lesions from low-grade lesions and for recognizing lesions with increased malignant potential. The progressive elevation of SOX2 expression across different stages of cervical pathology further supports its possible involvement in tumor development and progression, indicating potential relevance to future diagnostic and targeted therapeutic approaches.

From a translational perspective, SOX2 may be useful as an adjunct marker in diagnostic panels for cervical squamous lesions, particularly for recognizing lesions with increased malignant potential; however, its role in prognosis and treatment decision-making requires validation through larger studies with HPV correlation, longitudinal follow-up, and survival outcomes.

Overall, the findings indicate that SOX2 expression increased significantly from the nonmalignant cervical epithelium through premalignant lesions to invasive SCC, supporting its role in cervical carcinogenesis. However, its lack of significant association with SCC differentiation and FIGO stage suggests that SOX2 should not be interpreted as a standalone prognostic marker in the present cohort. Rather, SOX2 may be more useful as an adjunct biomarker reflecting malignant transformation and stemness-related biology. Its prognostic role requires validation in larger studies incorporating HPV status, p16/Ki-67 expression, lymphovascular invasion, recurrence, treatment response, and survival outcomes.

Certain limitations should be considered while interpreting these findings. The study was conducted at a single center with a relatively limited sample size, which may affect the generalizability of the results. Additionally, the cross-sectional design did not allow evaluation of long-term clinical outcomes or prognostic significance. Correlations with HPV infection status and other important biomarkers such as p16 and Ki-67 were also not explored. Future multicentric studies with larger cohorts and longitudinal follow-up are warranted to validate these observations and clarify the clinical significance of SOX2 expression in cervical lesions. Although the overall sample size provided adequate power for the primary comparison of SOX2 expression across diagnostic categories, some subgroup analyses were limited by small cell counts; therefore, these findings require confirmation in larger multicentric cohorts.

## Conclusions

The present study demonstrated a progressive increase in SOX2 expression from nonmalignant cervical epithelium to premalignant lesions and invasive SCC, indicating an association between SOX2 expression and cervical squamous lesion progression. Higher SOX2 expression was observed in premalignant and malignant lesions compared with nonmalignant cervical tissues, suggesting that SOX2 may reflect biological changes related to malignant transformation. However, SOX2 expression was not significantly associated with tumor differentiation or FIGO stage in this cohort. Therefore, the findings should be interpreted as associative rather than prognostic or therapeutic. Given the cross-sectional design, single-center setting, limited sample size, and absence of longitudinal outcome data, the present study does not establish SOX2 as a validated clinical biomarker or therapeutic target. Further multicentric studies with larger cohorts, HPV correlation, comparison with established markers such as p16 and Ki-67, and long-term follow-up, including recurrence, treatment response, and survival outcomes, are required to determine the diagnostic, prognostic, and therapeutic relevance of SOX2 in cervical squamous lesions.

## References

[1] Guo L, Liu Y, Zhang S, Liu W (2024). Case report: cutaneous metastasis of squamous cervical carcinoma: complete regression after molecular diagnosis. Front Immunol.

[2] Jariyal H, Gupta C, Bhat VS, Wagh JR, Srivastava A (2019). Advancements in cancer stem cell isolation and characterization. Stem Cell Rev Rep.

[3] Xu R, Yang WT, Zheng PS (2013). Coexpression of B-lymphoma Moloney murine leukemia virus insertion region-1 and sex-determining region of Y chromosome-related high mobility group box-2 in cervical carcinogenesis. Hum Pathol.

[4] Dayalan S, Raghavan V (2021). Comparative evaluation of SOX2 and p16 expression in intraepithelial neoplasia and invasive cancer of cervix. J Clin Diagn Res.

[5] Thakur A, Gupta B, Gupta A, Chauhan R (2015). Risk factors for cancer cervix among rural women of a hilly state: a case-control study. Indian J Public Health.

[6] Chang X, Zhang J, Huang C, Pang X, Luo Q, Zhang H, Zhang S (2015). Sex-determining region Y-related high mobility group box (SOX)-2 is overexpressed in cervical squamous cell carcinoma and contributes cervical cancer cell migration and invasion in vitro. Tumour Biol.

[7] Yang Z, Pan X, Gao A (2014). Expression of Sox2 in cervical squamous cell carcinoma. JBUON.

[8] Ji J, Wei X, Wang Y (2014). Embryonic stem cell markers Sox-2 and OCT4 expression and their correlation with WNT signal pathway in cervical squamous cell carcinoma. Int J Clin Exp Pathol.

[9] Bosch FX, Lorincz A, Muñoz N, Meijer CJ, Shah KV (2002). The causal relation between human papillomavirus and cervical cancer. J Clin Pathol.

[10] Gravitt PE, Winer RL (2017). Natural history of HPV infection across the lifespan: role of viral latency. Viruses.

[11] World Health Organization (2026). Comprehensive cervical cancer control: a guide to essential practice. https://www.who.int/publications/i/item/9789241548953.

[12] Dahiya N, Bachani D, Acharya AS (2017). Socio-demographic, reproductive and clinical profile of women diagnosed with advanced cervical cancer in a tertiary care institute of Delhi. J Obstet Gynaecol India.

[13] Pandey A, Chandra S, Nautiyal R, Shrivastav V (2018). Expression of p16(INK4a) and human papillomavirus 16 with associated risk factors in cervical premalignant and malignant lesions. South Asian J Cancer.

[14] Xu R, Chen L, Yang WT (2019). Aberrantly elevated Bmi1 promotes cervical cancer tumorigenicity and tumor sphere formation via enhanced transcriptional regulation of Sox2 genes. Oncol Rep.

[15] Kim BW, Cho H, Choi CH, Ylaya K, Chung JY, Kim JH, Hewitt SM (2015). Clinical significance of OCT4 and SOX2 protein expression in cervical cancer. BMC Cancer.

[16] Watanabe H, Suzuki A, Kobayashi M (2003). Analysis of temporal changes in the expression of estrogen-regulated genes in the uterus. J Mol Endocrinol.

[17] Fu HC, Chuang IC, Yang YC (2018). Low P16(INK4A) expression associated with high expression of cancer stem cell markers predicts poor prognosis in cervical cancer after radiotherapy. Int J Mol Sci.

[18] Cai CF, Tan GS, Yu Q, Luan F, Yu L, Wang Y (2015). Expression of SOX2 in cervical intraepithelial neoplasia and cervical cancer and its clinical significance. [Article in Chinese]. Nan Fang Yi Ke Da Xue Xue Bao.

[19] Moshi JM, Ummelen M, Broers JL (2022). SOX2 expression in the pathogenesis of premalignant lesions of the uterine cervix: its histo-topographical distribution distinguishes between low- and high-grade CIN. Histochem Cell Biol.

[20] Liu XF, Yang WT, Xu R, Liu JT, Zheng PS (2014). Cervical cancer cells with positive Sox2 expression exhibit the properties of cancer stem cells. PLoS One.

